# All-photonic quantum teleportation using on-demand solid-state quantum emitters

**DOI:** 10.1126/sciadv.aau1255

**Published:** 2018-12-14

**Authors:** Marcus Reindl, Daniel Huber, Christian Schimpf, Saimon F. Covre da Silva, Michele B. Rota, Huiying Huang, Val Zwiller, Klaus D. Jöns, Armando Rastelli, Rinaldo Trotta

**Affiliations:** 1 Institute of Semiconductor and Solid State Physics, Johannes Kepler University, 4040 Linz, Austria.; 2 Department of Physics, Sapienza University of Rome, 00185 Rome, Italy.; 3 Department of Applied Physics, Royal Institute of Technology, 106 91 Stockholm, Sweden.; 4 Linz Institute of Technology, Johannes Kepler University, 4040 Linz, Austria.

## Abstract

All-optical quantum teleportation lies at the heart of quantum communication science and technology. This quantum phenomenon is built up around the nonlocal properties of entangled states of light that, in the perspective of real-life applications, should be encoded on photon pairs generated on demand. Despite recent advances, however, the exploitation of deterministic quantum light sources in push-button quantum teleportation schemes remains a major open challenge. Here, we perform an important step toward this goal and show that photon pairs generated on demand by a GaAs quantum dot can be used to implement a teleportation protocol whose fidelity violates the classical limit (by more than 5 SDs) for arbitrary input states. Moreover, we develop a theoretical framework that matches the experimental observations and that defines the degree of entanglement and indistinguishability needed to overcome the classical limit independently of the input state. Our results emphasize that on-demand solid-state quantum emitters are one of the most promising candidates to realize deterministic quantum teleportation in practical quantum networks.

## Teaser

Photons generated on-demand by artificial atoms are used to demonstrate quantum teleportation.

## INTRODUCTION

The advent of quantum technologies in everyday life requires the realization of a quantum network, allowing sharing of quantum information between different quantum computation nodes (*1*). Independently of the material platform used for the first quantum computers, the network will rely on sources of single photons generated on demand. For worldwide quantum networks, however, this is not quite sufficient, as the no-cloning theorem hinders the amplification of the transmitted signals (*2*). Linking quantum network nodes located more than a few hundred kilometers apart thus requires the implementation of quantum teleportation protocols (*3*). After the first experimental demonstrations of quantum teleportation (*4*, *5*), quantum repeater protocols (*6*–*8*) have been developed to increase the range of quantum communication. In contrast to the commonly used DLCZ (for Duan, Lukin, Cirac, and Zoller) scheme (*7*) that generates entanglement via quantum interference, higher data rates can be achieved using entangled photon pair sources in a quantum relay configuration (*9*, *10*) in conjunction with quantum memories. Therefore, substantial research has been carried out to find the most suitable source of entangled photon pairs to realize these quantum relays. Among others (*11*), semiconductor quantum dots (QDs) are emerging as near-optimal sources of indistinguishable single photons (*12*, *13*), as well as on-demand entangled photon pairs (*14*). However, quantum teleportation based on these entangled photon sources was, so far, only preliminarily investigated for the InGaAs QD system (*15*) and, up to now, verified for arbitrary input states only in laser heralded teleportation schemes (*16*–*18*). Moreover, in none of these works were photons generated on demand, one of the key requirements for real-life applications that rely on push-button quantum teleportation schemes. Here, we show the first quantum teleportation of on-demand generated photon polarization states by exploiting single and entangled photons from previously unknown GaAs QDs (*19*). We implement a teleportation protocol whose fidelity overcomes the classical limit for a full set of orthogonal input states as required to truly verify quantum teleportation. In addition, we carry out the experiments using several different QDs so as to demonstrate the general relevance of our result. Last, we build up a theoretical model that identifies the requirements needed to successfully perform quantum teleportation with semiconductor quantum light sources. Our results emphasize the potential of semiconductor QDs as near-ideal sources for all-optical quantum relays.

## RESULTS

**Fig. 1:**
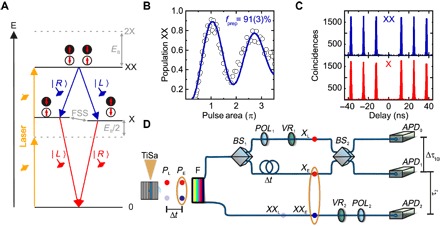
On-demand photon source and quantum teleportation setup. (**A**) The radiative recombination of XX-X states provides two photons entangled in polarization if the energetic splitting of the X state, the fine structure splitting (FSS), is sufficiently low. The on-demand generation occurs via a resonant laser tuned to half the energy of the XX state. *E*_B_ indicates the XX binding energy. (**B**) Population of the XX state as a function of the pulse area. The experimental data (circles) are modeled as an exponentially damped sine-squared function (purple curve) to determine the depicted preparation fidelity. (**C**) The autocorrelation measurements for the XX and X transition of a representative QD. (**D**) The experimental setup for quantum teleportation. A pulsed laser [titanium sapphire (TiSa)] is used to excite two times the QD, which then emits an early pair (*P*_E_) and a late pair (*P*_L_) of entangled photons separated by Δ*t* in time. The XX and X photons are then spectrally separated by a filter (F). The early *X*_E_ and late *X*_L_ pass a HOM Mach-Zehnder consisting of two beam splitters (BSs), performing the Bell state measurement. Polarizers (POLs) and variable retarders (VRs) are used to define the *X*_L_ input state and *XX*_E_ detection state accordingly. The three-photon correlation measurement is then recorded as a function of arrival times τ with avalanche photodiodes (APDs).

We start out by introducing our photon emitter as a pure and on-demand source of single and entangled photons: highly symmetric GaAs/AlGaAs QDs fabricated via the droplet etching method (*20*) (see Materials and Methods). The QDs are embedded in a low-Q distributed Bragg reflector (DBR) cavity featuring a solid immersion lens to enhance the single-photon light collection efficiency up to values around 12%. The first requirement in our experiment is to ensure the on-demand generation of photon pairs, which are generated via the XX (biexciton)–X (exciton) cascade (*21*) shown in Fig. 1A. Therefore, we use a pulsed two-photon excitation (TPE) scheme (*14*, *22*) to directly populate the XX state by means of a controlled π pulse with fidelity of *f*_prep_ = 0.91(0.03) (Fig. 1B). The deviation from *f*_prep_ = 1 can be mainly understood by electron-phonon interactions, as reported in the literature (*23*, *24*). It is important to emphasize that the TPE scheme is fundamental to minimize re-excitation processes (*25*), as proven by the excellent single-photon purity of our source (Fig. 1C). In particular, we observe second-order coherences in the range of 0.004(0.002) ≤ $g_{XX}^{2}(0)$ ≤ 0.009(0.002) and 0.009(0.001) ≤ $g_{X}^{2}(0)$ ≤ 0.034(0.005) for XX and X transitions featuring average lifetimes of *T*_1,*XX*_ ≈ 140(7) ps and *T*_1,*X*_ ≈ 265(10) ps, respectively (see fig. S1 and table S1). While our sample can therefore generate pure single and entangled photons on demand, photonic structures featuring near-unity extraction efficiencies are needed to realize a fully deterministic entangled photon source. This is still an open challenge, but ongoing efforts (*26*, *27*) suggest that this will be possible in the near future.

Next, we discuss the experimental setup used to perform quantum teleportation with our on-demand generated pure single photons (see Fig. 1D). A pulsed laser generates two pairs of entangled photons temporally spaced by Δ*t* = 2 ns during every duty cycle of 12.5 ns. The generated XX and X photons are spectrally separated using a filter with bandwidth 150 times larger than the QD transition linewidth, that is, we do not spectrally filter any photons of the raw QD emission spectrum. The two consecutive XX photons are sent directly to an APD connected to the correlation electronics. The two consecutive X photons are instead launched to an unbalanced Mach-Zehnder interferometer necessary to overlap them at *BS*_2_, which is used for the Bell state measurement. For clarity, Fig. 1D shows only the configuration in which the early photon (*X*_E_) takes the long path, while the late photon (*X*_L_) takes the short path. In this case, the two X photons arrive at the same time at *BS*_2_ required for the quantum interference. All the other possibilities correspond to X photons impinging on *BS*_2_ at different times. While these photons are not relevant for the teleportation scheme, they do produce correlations visible in the experimental data, as discussed in more detail later in the text. In the teleportation protocol used in this work, we therefore use three photons, the entangled *X*_E_-*XX*_E_ two-photon state and the single *X*_L_ photon, the latter being used to encode the polarization state to be teleported onto *XX*_E_. This is achieved by placing a linear polarizer (*POL*_1_) and a waveplate (*VR*_1_) (λ/2 or λ/4) to define the teleportation input state. Upon successful coalescence of the two X photons, we then expect to observe the reconstruction of the polarization state on the early *XX*_E_ by setting the appropriate polarization detection basis (*VR*_2_ and *POL*_2_).

The teleportation dynamics can be understood by taking into account the Bell state measurement (performed on the *X*_E_ and the *X*_L_ photons) and the entangled two-photon (*X*_E_ and *XX*_E_) state. The detected Bell state is chosen to be |ψ^−^〉, as it is easily distinguishable from the other emerging Bell states upon quantum interference (see the Theory section in the Supplementary Materials). For the given experimental implementation, |ψ^−^〉 exclusively yields a single photon at each of the output modes of *BS*_2_ (left *l*^†^ and right *r*^†^) simultaneously and causes a click event on both photodetectors

$${|\psi^{-}\rangle }_{l,r}=\frac{1}{\sqrt{2}}({|H\rangle }_{l^{†}}{|V\rangle }_{r^{†}}+{|V\rangle }_{l^{†}}{|H\rangle }_{r^{†}})$$ (1)

where |*H*〉 and |*V*〉 are linear-polarized states. The polarization-entangled state of the QD transition cascade can be interpreted as an evolving |φ^+^〉 Bell state if examined in the linear polarization basis

$${|\varphi^{+}\rangle }_{X_{E},XX_{E}}(t)=\frac{1}{\sqrt{2}}({|H\rangle }_{X_{E}}{|H\rangle }_{XX_{E}}+e^{-\frac{iFSSt}{\hbar }}{|V\rangle }_{X_{E}}{|V\rangle }_{XX_{E}})$$ (2)

where we account for the time evolution of the entangled state arising from an energetic splitting between the two bright X states, the so-called FSS (see Fig. 1A). The arbitrarily chosen input state of the late exciton ${|\psi \rangle }_{X_{L}}=a|H\rangle +b|V\rangle$ is then used to define the combined wave function of the three-photon experiment

$$\begin{array}{cc} {|\psi \rangle }_{X_{L},X_{E},XX_{E}}(t)= & {|\psi \rangle }_{X_{L}}⊗{|\psi \rangle }_{X_{E},XX_{E}}(t)=\\ = & \frac{1}{2}{|\varphi^{+}\rangle }_{X_{L},X_{E}}⊗(a{|H\rangle }_{XX_{E}}+e^{\delta (t)}b{|V\rangle }_{XX_{E}})\\ & +\frac{1}{2}{|\varphi^{-}\rangle }_{X_{L},X_{E}}⊗(a{|H\rangle }_{XX_{E}}-e^{\delta (t)}b{|V\rangle }_{XX_{E}})\\ & +\frac{1}{2}{|\psi^{+}\rangle }_{X_{L},X_{E}}⊗(b{|H\rangle }_{XX_{E}}+e^{\delta (t)}a{|V\rangle }_{XX_{E}})\\ & +\frac{1}{2}{|\psi^{-}\rangle }_{X_{L},X_{E}}⊗(b{|H\rangle }_{XX_{E}}-e^{\delta (t)}a{|V\rangle }_{XX_{E}}) \end{array}$$ (3)

where we now focus only on the specific three-photon output governed by the chosen |ψ^−^〉 detection (see the Theory section in the Supplementary Materials). If the Bell state measurement is successful, that is, we detect the |ψ^−^〉 state through two simultaneous clicks at *APD*_0_ and *APD*_1_, then the polarization of the output photon ${|\psi \rangle }_{XX_{E}}$ reads as

$${|\psi \rangle }_{XX_{E}}^{\psi^{-}}=\sigma_{y}(t){|\psi \rangle }_{X_{L}}\;\text{with}\sigma_{y}(t):=(\begin{array}{ccc} 0 & 1\\ -e^{-\frac{iFSSt}{\hbar }} & & 0 \end{array})$$ (4)

The teleportation fidelity can then be calculated as (*28*)

$$f_{T}=Tr[\rho_{XX_{E}}\sigma_{y}{|\psi \rangle }_{X_{L}}{\langle \psi |}_{X_{L}}\sigma_{y}^{†}]$$ (5)

where $\rho_{XX_{E}}$ is the single-photon density matrix of the reconstructed state.

**Fig. 2:**
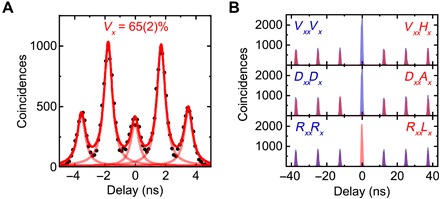
Indistinguishability and degree of entanglement of QD1. (**A**) The two-photon interference measurement using copolarized settings. The histograms envelope function (bold red) is the sum of five Lorentzian peaks fitted to the HOM quintuplet. The resulting raw two-photon interference visibility is *V*_*X*_ = 65(2) %. (**B**) XX-X cross-correlation measurements for different polarization detection bases: rectilinear (*V*, *H*), diagonal (*D*, *A*), and circular basis (*R*, *L*).

Experimentally, the teleportation fidelity depends on two main parameters: (i) the Hong-Ou-Mandel (HOM) visibility of the X photons, which strongly affects the success ratio to trigger an actual |ψ〉^−^ detection event, and (ii) the initial entanglement fidelity to the expected Bell state |φ〉^+^. The HOM visibility parameter (i) can be probed using co-polarized settings for *X*_E_ and *X*_L_ photons using a relative delay of approximately Δ*t* = 2 ns (for this test, a polarizer is placed also on the long path) and can be quantified looking at the central peak of the resulting histogram quintuplet (Fig. 2A). In particular, the ratio of the central peak to the average of the next neighbor side peaks is related to the two-photon interference visibility, which, for QD1, is 65(2)%. This value is the raw indistinguishability, and it is not corrected for any experimental imperfections (specifically the splitting ratio of *BS*_2_, the overlap of the two input modes, and the nonzero $g_{X}^{2}(0)$). By taking these factors into account, we would get a HOM visibility $V_{X}^{\text{corr}}=71(3)\%$. It is also important to emphasize that we do not temporally postselect emission events shortly after the excitation (on time scales shorter than the X lifetime). While this is routinely done to partially avoid dephasing and improve the visibility, it reduces substantially the effective brightness of the source and the on-demand character of the photon generation, one of the main advantages of QDs compared with other nonclassical light sources. For this reason, we prefer to avoid postselecting on the relevant peak and perform the experiment using all the photons that arrive at the BS in the configuration of Fig. 1D (*X*_E_ and *X*_L_ impinge on the BS at the same time). We assume that enhancing the indistinguishability of droplet-etched GaAs QDs toward unity can be done by the optimization of QD growth parameters to engineer the dephasing arising from electron-phonon interaction (*29*) and embedding the emitters in photonic structures, leading to enhanced spontaneous emission rates through the Purcell effect (*30*).

The other relevant parameter, the entanglement fidelity (ii), is measured by a set of XX-X cross-correlation measurements in different polarization basis. Using the formula (*31*)

$$f_{E}^{|\varphi^{+}\rangle }=\frac{1+C_{\text{HV}}+C_{\text{DA}}-C_{\text{RL}}}{4}$$ (6)

where the $C_{ij}=\frac{g_{ii}^{\left(2\right)}(0)-g_{ij}^{\left(2\right)}(0)}{g_{ii}^{\left(2\right)}(0)+g_{ij}^{\left(2\right)}(0)}$ represent normalized correlation measurements in the XX base *i* and X base *j*, we find that the fidelity to the expected |φ^+^〉 state is *f*_E_ = 0.925(0.003) at a finite excitonic FSS of 1.15(0.2) μeV (Fig. 2B). This demonstrates that our GaAs QDs can deliver high-quality entangled photons, which can potentially reach fidelities for FSS = 0 as large as 98% (*32*).

**Fig. 3:**
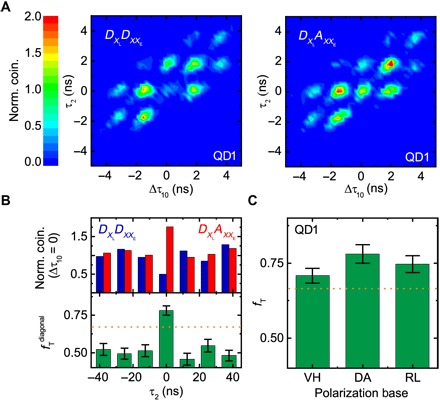
Measurement of the teleportation fidelity using QD1. (**A**) Normalized third-order correlation of a D-polarized X input state for copolarized (left) and cross-polarized (right) detection of the XX photons. (**B**) Integrated coincidences for both detection bases for different excitation cycles (top) and the corresponding calculated teleportation fidelity (bottom). (**C**) Teleportation fidelities for a full set of orthogonal input states. The classical limit is highlighted as a dashed orange line.

Having precharacterized the photons emitted by QD1, we now test its suitability as a light source for quantum teleportation between on-demand generated true single photons. We record the third-order correlations for different input state polarizations in the previously described experimental setup. To build up the coincidence histogram, we merged the arrival times of the X photons on the individual detectors to a corresponding time difference Δτ_10_, meaning that only at Δτ_10_ = 0 can we expect a successful |ψ〉^−^ detection. Furthermore, we set the relative arrival time τ_2_ of the teleported *XX*_E_ photon to τ_2_ = 0. The consecutive double excitation of the QD should therefore provide a set of 12 possible time correlations, iterating every 12.5 ns. Exemplary for one measurement, we show in Fig. 3A the result for the experimentally defined ${|\psi \rangle }_{X_{L}}={|D\rangle }_{X_{L}}$ input state around Δτ_10_ = τ_2_ = 0 for co- and cross-polarized XX detection bases (a full set of measurements showing the third-order correlation for long time scales is reported in fig. S1). As mentioned above, we do not discuss the side peaks in detail here as they originated from photons not interfering at *BS*_2_ or stem from *XX*_L_ photons and are therefore not relevant to the teleportation protocol discussed in this work. The data are normalized to the uncorrelated side peak coincidences, that is, those corresponding exactly to the same type of correlation events occurring at time scales longer than 12.5 ns. Taking into account that the measured FSS is very close to zero for QD1, the peak at zero time delay in Fig. 3A shows the behavior predicted by Eq. 4, that is, we measure a strong correlation when there is a flip in polarization, as expected for the σ_*y*_ transformation of the input state. The fidelity of teleportation can be estimated from the (normalized) detection probabilities of the co- and cross-polarized third-order correlations

$$f_{T}^{\text{diagonal}}=\frac{g_{|A\rangle }^{\left(3\right)}(0)}{g_{|A\rangle }^{\left(3\right)}(0)+g_{|D\rangle }^{\left(3\right)}(0)}$$ (7)

and yields a value as high as 78(3)% (Fig. 3B). As required for quantum teleportation, we performed similar studies for varying input state polarizations (see fig. S2), and we report the calculated teleportation fidelities in Fig. 3C. Here, we decided to resemble three different input states equally spaced on the Bloch sphere to conclude on the average teleportation fidelity since any general state can be then described by a linear combination of these three states (*33*). We violate the average classical limit of $\frac{2}{3}$ independently of the chosen input state, meaning that we can exclude any bias stemming from the experimental setup. This is particularly important; as in fig. S3, we demonstrate that, when only classically correlated photons are used in the teleportation experiment, it is not possible to overcome the classical limit for a complete set of input states representing the full Bloch sphere, that is, it is not possible to claim a successful quantum teleportation without resorting to assumptions on the experimental apparatus, as actually done in previous experiments (*15*). Therefore, the experimental results shown in Fig. 3 represent a striking evidence of true quantum teleportation performed with solely QD photons. The average fidelity is 75(2)%, a result achieved without temporal postselection on the relevant peak, background subtraction, nor postprocessing of the measured data. In other words, the measured fidelity corresponds to the raw data.

**Fig. 4:**
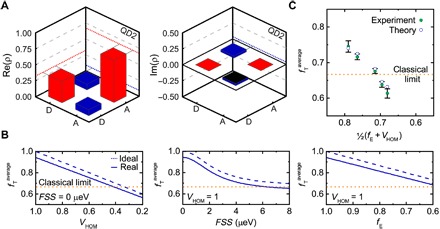
Single-qubit tomography and theoretical modeling of experimental data. (**A**) Real and imaginary parts of the measured density matrix of the quantum teleportation using QD2. The reference frame is chosen with respect to the diagonal input state. (**B**) Calculated average teleportation fidelity based on our model. The change in teleportation fidelity is shown for vanishing FSS (perfect entanglement fidelity) and varying visibility and for perfect visibility and varying FSS. The teleportation fidelity as a function of the entanglement fidelity is additionally calculated from the two-photon state’s evolution using the average X lifetime of $T_{1_{X}}=265(10)$ ps. Each of the panels contains the expected ideal behavior (dashed line) compared with the real experimental conditions (solid line), considering polarization-dependent, relative phase shifts introduced by the optical setup. (**C**) The average teleportation fidelity of all measured QDs (green dot) and their predicted theoretical value (blue circle).

After having verified that QD photons can be used in a teleportation protocol with arbitrary input states, we extend our study to different QDs. We do so for two main reasons: first, to prove that our result is general and not QD specific, and, second, to investigate the degree of entanglement and indistinguishability needed to successfully overcome the classical limit. Therefore, we measured the entanglement fidelity and two-photon interference visibility of many different QDs (see table S2) and built up a theoretical model to reproduce the experimental teleportation fidelities as a function of these two parameters. To estimate the average teleportation fidelity according to Eq. 5, we still miss a formalism for the density matrix of the output photon, which fully reads

$$\rho_{XX_{E}}=\int_{t_{\text{min}}}^{t_{\text{max}}}P(t)\sum_{B}p_{|B\rangle }{|\psi \rangle }_{XX_{E}}^{B}{\langle \psi |}_{XX_{E}}^{B}(t)dt$$ (8)

where $P(t)=\frac{1}{T_{1_{X}}}e^{\frac{-t}{T_{1_{X}}}}$ is the probability distribution function of the entangled photon pair within the X lifetime $T_{1_{X}}$, while the *p*_|*B*〉_ represents the success of Bell state *B* (|φ^+^〉,|φ^−^〉,|ψ^+^〉, or |ψ^−^〉) detection and is related to the HOM visibility (see the Theory section in the Supplementary Materials). Since we integrate in a range much larger than the X lifetime and do not apply temporal filtering, we can assume the time range *t*_min_ = 0 and *t*_max_ = ∞ in our calculations. The probability distribution function *P*(*t*) combined with the evolution of the entangled state, as introduced for the output photon in Eq. 4, scales [in good approximation (*32*)] with the entanglement fidelity of the ${|\varphi^{+}\rangle }_{X_{E},XX_{E}}(t)$ two-photon state. Last, to correctly compare the measured teleportation fidelities with the prediction of Eq. 5, we must take into account possible polarization rotations induced by our experimental setup. For this reason, we performed a full tomography on a second QD (QD2) so as to obtain the single-qubit density matrices [see the Theory section in the Supplementary Materials and (*34*)]. The real and imaginary parts of the experimental density matrix for a diagonal input state (plotted in the respective eigensystem) are depicted in Fig. 4A. From this matrix, we can extract the teleportation fidelity, which turns out to be 75.5(1.5)%. By performing the measurement for all the other input states (see fig. S4), we find an average fidelity of 71.8(0.9)%, which is in excellent agreement with the previous method based on Eq. 7, yielding 71.6(0.8)%. The imaginary part of these qubit matrices discloses the existence of relative phase shifts between ${|\psi \rangle }_{X_{L}}$ and ${|\varphi \rangle }_{XX_{E}}$ photons that are introduced by polarization-dependent imperfections of the optical elements [scattered laser photons lowering the *g*^(2)^(0) should also be considered, but they have a minor effect here]. These measurements allow us to take into account imperfections in our simulations by evaluating the Stokes vectors *S*_*X*_ and *S*_*Y*_ from the off-diagonal matrix elements and to calculate the angular offset with respect to *S*_*Z*_ (the expected eigensystem). The obtained angle is then simply applied as a rotation matrix on the respective input state to account for the said phase shift. We can now calculate how the teleportation fidelity evolves with the FSS and *V*_HOM_, with and without the imperfections related to the experimental setup (Fig. 4B). Our model shows that at FSS = 0, visibilities above 50% are needed to overcome the classical limit. The teleportation fidelity is instead more tolerant to the FSS (at *V*_HOM_ = 1), which can be as large as 5 μeV. This evidence is related to the fact that the lifetime of the X state is rather short in our QDs [$T_{1_{X}}=265(10)$ ps], thus leading to a rapidly decaying probability density function in comparison to the time evolution of the entangled state. To verify the prediction of our model, we measured several QDs and compared the average teleportation fidelities as a function of photon indistinguishability and entanglement fidelity in Fig. 4C (in the model, the lifetime for each QD is set to the experimental values). For the sake of simplicity, data are shown as a function of 1/2(*f*_E_ + *V*_HOM_), although we stress that *V*_HOM_ has a stronger impact on the resulting teleportation fidelity compared with *f*_E_. We observe an excellent quantitative agreement between experiments and theory, meaning that the theory is able to grasp the physics underlying the measured values of teleportation fidelities on semiconductor QDs. The small discrepancy is mainly attributed to the uncertainty of up to 0.3 μeV when determining the FSS.

## DISCUSSION

Our demonstration of quantum teleportation between on-demand generated entangled photon pairs marks an important step toward solid-state–based all-photonic quantum relays. We were able to demonstrate quantum teleportation using solely photons generated on demand by a solid-state quantum emitter. Moreover, we developed a theoretical framework that can be used as a general guideline to achieve successful teleportation in any QD-based system. Our work also pinpoints the next challenges that have to be overcome to finally use QDs in real-life applications, where fully deterministic photon sources and unconditional teleportation protocols are necessary: (i) the development of efficient broadband cavities to increase the photon pair emission rate, (ii) the improvement of the two-photon interference visibility, (iii) the integration of QDs in devices enabling the application of external fields for engineering the photon emission properties, and (iv) the establishment of a full Bell state measurement protocol to maximize the teleportation rate. Of these challenges, (i) and (ii) could be addressed using photonic cavities that boost the flux of QD photons (*27*, *35*) and provide enough Purcell enhancement to improve the photon indistinguishability (*36*). The third one (iii) is instead needed to interface photons emitted by dissimilar QDs (*37*) in the scope of remote teleportation protocols (*38*, *39*). The last step (iv) can be potentially addressed via hyperentanglement schemes (*40*). Developing a device that fulfills all these points simultaneously to establish push-button teleportation protocols is surely a grand challenge. Nonetheless, it is definitively worth the effort, as it can bring about a revolution in the field of quantum communication and quantum networks.

## MATERIALS AND METHODS

### Materials

The QD layer was obtained by Al droplet etching on Al_0.4_Ga_0.6_As, followed by deposition of 2-nm GaAs. This technique allows the fabrication of highly symmetric QDs. The QD layer was placed at the center of a λ cavity mode of λ/2-thick (123 nm) layer of Al_0.4_Ga_0.6_As sandwiched between two λ/4-thick (60 nm) Al_0.2_Ga_0.8_As layers. The cavity sits on top of a DBR made of nine pairs of λ/4-thick Al_0.95_Ga_0.05_As (70 nm) and Al_0.2_Ga_0.8_As layers and below two pairs of the same material combination. A 4-nm-thick GaAs protective layer completes the structure. The limited number of pairs used for the DBR mirrors does not yield any measurable Purcell enhancement but an increase in light extraction efficiency. Last, a solid immersion lens was placed on top of the grown sample to enhance the external collection efficiency to values up to 12%.

### Methods

All the measurements were performed at sample temperatures of 10 K in helium flow cryostats. The resonant excitation was done with a titanium sapphire femtosecond laser featuring a bandwidth of 100 fs and a repetition rate of 80 MHz passing a 4f pulse-shaper setup to form picosecond pulses (10 ps). The laser was focused via an objective (numerical aperture of 0.42) onto the sample. We used tunable notch filters in transmission with a bandwidth of 0.4 nm to reject scattered laser light. Before fiber coupling of the QD signal, the light was guided to and spectrally filtered by another set of tunable notch filters used in reflection to separate X and XX emission. The signal was then detected either by a charge-coupled device camera or—during photon correlation spectroscopy—by APDs connected to the correlation electronics. The temporal resolution of these detectors is about 500 ps (40 dark counts per second). Combinations of λ/2, λ/4 and fixed polarizers were properly placed to estimate the entanglement or teleportation fidelity. The two-photon interference experiments rely on a single-mode fiber BS with reflectance *R* = 49.0(1)%, transmittance *T* = 51.0(1)%, and mode overlap 1 - ε = 96(1)%. The measurements of the FSS were carried out with a rotating λ/2 waveplate in front of a fixed polarizer placed at the entrance of a grating spectrometer (1800 lines/mm). Using Gaussian fitting of both the X and XX lines, the FSS can be measured with sub-micro-electronvolt resolution. Lifetime measurements were performed using another APD featuring a temporal resolution of 50 ps.

## Supplementary Material

http://advances.sciencemag.org/cgi/content/full/4/12/eaau1255/DC1

## SUPPLEMENTARY MATERIALS

Supplementary material for this article is available at http://advances.sciencemag.org/cgi/content/full/4/12/eaau1255/DC1 Theory Fig. S1. Lifetime and full third-order correlation. Fig. S2. Teleportation measurements on QD1. Fig. S3. Classical teleporter. Fig. S4. Single-qubit density matrices for QD2. Table S1. Lifetimes of measured QDs. Table S2. Compiled experimental and theoretical results of all QDs under investigation.
