# Supplementary material for: All-photonic quantum teleportation using on-demand solid-state quantum emitters
Source: Sci Adv. 2018 Dec 14;4(12):eaau1255. doi: 10.1126/sciadv.aau1255 (PMC6294597; doi:10.1126/sciadv.aau1255)
Supplement: http://advances.sciencemag.org/cgi/content/full/4/12/eaau1255/DC1 [file supp_4_12_eaau1255__index.html]

Science Advances | Science Advances

## Supplementary Materials

**This PDF file includes:**

- Theory
- Fig. S1. Lifetime and full third-order correlation.
- Fig. S2. Teleportation measurements on QD1.
- Fig. S3. Classical teleporter.
- Fig. S4. Single-qubit density matrices for QD2.
- Table S1. Lifetimes of measured QDs.
- Table S2. Compiled experimental and theoretical results of all QDs under investigation.

Download PDF

**Files in this Data Supplement:**

- Adobe PDF - aau1255\_SM.pdf
