# Supplementary material for: All-photonic quantum teleportation using on-demand solid-state quantum emitters
Source: Sci Adv. 2018 Dec 14;4(12):eaau1255. doi: 10.1126/sciadv.aau1255 (PMC6294597; doi:10.1126/sciadv.aau1255)
Supplement: http://advances.sciencemag.org/cgi/content/full/4/12/eaau1255/DC1 [file aau1255_SM.pdf]

Science Advances
AAAS

advances.sciencemag.org/cgi/content/full/4/12/eaau1255/DC1

Supplementary Materials for

All-photonic quantum teleportation using on-demand solid-state quantum emitters

Marcus Reindl*, Daniel Huber, Christian Schimpf, Saimon F. Covre da Silva, Michele B. Rota, Huiying Huang, Val Zwiller, Klaus D. Jöns, Armando Rastelli, Rinaldo Trotta*

*Corresponding author. Email: marcus.reindl@jku.at (M.R.); rinaldo.trotta@uniroma1.it (R.T.)

Published 14 December 2018, *Sci. Adv.* **4**, eaau1255 (2018)
DOI: 10.1126/sciadv.aau1255

This PDF file includes:

Theory
Fig. S1. Lifetime and full third-order correlation.
Fig. S2. Teleportation measurements on QD1.
Fig. S3. Classical teleporter.
Fig. S4. Single-qubit density matrices for QD2.
Table S1. Lifetimes of measured QDs.
Table S2. Compiled experimental and theoretical results of all QDs under investigation.

Theory

Bell state measurement

In general, the unitary transformations of the creation operators $\hat{a}^\dagger$ (at fixed polarization σ) of the incoming photon modes l and r with respect to the outcoming photon modes $l^\dagger$ and $r^\dagger$ passing a 50/50 BS (π-shift upon reflection) read as follows

$$\hat{a}^\dagger_{l,\sigma} \rightarrow \frac{\hat{a}^\dagger_{l^\dagger,\sigma} + i\hat{a}^\dagger_{r^\dagger,\sigma}}{\sqrt{2}} \qquad \hat{a}^\dagger_{r,\sigma} \rightarrow \frac{i\hat{a}^\dagger_{l^\dagger,\sigma} + \hat{a}^\dagger_{r^\dagger,\sigma}}{\sqrt{2}} \tag{1}$$

These transformations applied to single photon input states ($|1\rangle_l$ and $|1\rangle_r$) allows to obtain the well known quantum superposition state

$$|1\rangle_l |1\rangle_r = \frac{1}{2}(\hat{a}^\dagger_{l,\sigma} + i\hat{a}^\dagger_{r,\sigma})(\hat{a}^\dagger_{r,\sigma} + i\hat{a}^\dagger_{l,\sigma}) |0\rangle_l |0\rangle_r = \frac{1}{\sqrt{2}}(|2\rangle_{l^\dagger} |0\rangle_{r^\dagger} + |0\rangle_{l^\dagger} |2\rangle_{r^\dagger}) \tag{2}$$

Let's now consider the input photons to be in one of the maximally entangled Bell states. Using the creation operators we can rewrite the transformation as

$$\begin{aligned}
|\phi^+\rangle_{lr} &= \frac{1}{\sqrt{2}} \left(\hat{a}^\dagger_{l,H}\hat{a}^\dagger_{r,H} + \hat{a}^\dagger_{l,V}\hat{a}^\dagger_{r,V}\right) |0\rangle_l |0\rangle_r \\
|\phi^-\rangle_{lr} &= \frac{1}{\sqrt{2}} \left(\hat{a}^\dagger_{l,H}\hat{a}^\dagger_{r,H} - \hat{a}^\dagger_{l,V}\hat{a}^\dagger_{r,V}\right) |0\rangle_l |0\rangle_r \\
|\psi^+\rangle_{lr} &= \frac{1}{\sqrt{2}} \left(\hat{a}^\dagger_{l,H}\hat{a}^\dagger_{r,V} + \hat{a}^\dagger_{l,V}\hat{a}^\dagger_{r,H}\right) |0\rangle_l |0\rangle_r \\
|\psi^-\rangle_{lr} &= \frac{1}{\sqrt{2}} \left(\hat{a}^\dagger_{l,H}\hat{a}^\dagger_{r,V} - \hat{a}^\dagger_{l,V}\hat{a}^\dagger_{r,H}\right) |0\rangle_l |0\rangle_r
\end{aligned} \tag{3}$$

Using Equation (1) we can calculate how each Bell state transforms after the BS

$$
\begin{aligned}
|\chi\rangle_{\phi^+} &= \frac{1}{2}\left(|HH\rangle_{l^\dagger} + |VV\rangle_{l^\dagger} + |HH\rangle_{r^\dagger} + |VV\rangle_{r^\dagger}\right) \\
|\chi\rangle_{\phi^-} &= \frac{1}{2}\left(|HH\rangle_{l^\dagger} - |VV\rangle_{l^\dagger} + |HH\rangle_{r^\dagger} + |VV\rangle_{r^\dagger}\right) \\
|\chi\rangle_{\psi^+} &= \frac{1}{\sqrt{2}}\left(|HV\rangle_{l^\dagger} + |HV\rangle_{r^\dagger}\right) \\
|\chi\rangle_{\psi^-} &= \frac{1}{\sqrt{2}}\left(|H\rangle_{l^\dagger}|V\rangle_{r^\dagger} + |V\rangle_{l^\dagger}|H\rangle_{r^\dagger}\right)
\end{aligned}
\tag{4}
$$

In an ideal teleportation experiment, the states described in Equation (4) will emerge from the BS with equal probability. Out of the 4 states, the last one is the only one that provides a single photon at each of the output ports and, therefore, can be easily detected by recording simultaneous coincidences between two APDs placed right after the BS. While this simplifies the experimental set-up, it also reduces the number of three-fold coincidence in a teleportation experiment. It is also worth noting that the discussion above holds for completely indistinguishable photons. In the next section, we discuss the more general case where photons are only partially indistinguishable.

Teleportation fidelity

The overlap in Hilbert space of two quantum states, hence fidelity, gives an approximation of the successful state reconstruction.The density matrix of the input photon is an experimentally

prepared single photon state whose polarization are to be teleported, therefore simply reads as $\rho_{X_L} = |\psi\rangle_{X_L} \langle\psi|_{X_L}$. The statistically mixed density matrix of the output photon ρ_{XX_E} essentially depends on the outcome of a Bell state measurement for the three-photon quantum superposition $|\psi\rangle_{X_L XX_E XX_E}$. The arbitrary input wavefunction in the linear-polarized frame may be expressed as

$$|\psi\rangle_{X_L} = a\,|H\rangle + b\,|V\rangle \tag{5}$$

The other two photons are in the known polarization-entangled two-photon state emerging from the QD transition cascade

$$|\psi\rangle_{X_E,XX_E}(t) = \frac{1}{\sqrt{2}}\left(|H\rangle_{X_E}|H\rangle_{XX_E} + e^{\delta(t)}|V\rangle_{X_E}|V\rangle_{XX_E}\right) \tag{6}$$

and accounts for the evolution of the superposition state in the presence of a finite fine structure splitting $\delta(t) = -\frac{iFSSt}{\hbar}$. Using the following equations

$$\begin{aligned}
|HH\rangle_{X_L,X_E} &= \frac{1}{\sqrt{2}}\left(|\phi^+\rangle_{X_L,X_E} + |\phi^-\rangle_{X_L,X_E}\right)\\
|VV\rangle_{X_L,X_E} &= \frac{1}{\sqrt{2}}\left(|\phi^+\rangle_{X_L,X_E} - |\phi^-\rangle_{X_L,X_E}\right)\\
|HV\rangle_{X_L,X_E} &= \frac{1}{\sqrt{2}}\left(|\psi^+\rangle_{X_L,X_E} + |\psi^-\rangle_{X_L,X_E}\right)\\
|VH\rangle_{X_L,X_E} &= \frac{1}{\sqrt{2}}\left(|\psi^+\rangle_{X_L,X_E} - |\psi^-\rangle_{X_L,X_E}\right)
\end{aligned} \tag{7}$$

the wavefunction of the whole quantum system reads

$$\begin{aligned}
|\psi\rangle_{X_L,X_E,XX_E}(t) = |\psi\rangle_{X_L} \otimes |\psi\rangle_{X_E,XX_E}(t) = &\\
= \frac{1}{2}|\phi^+\rangle_{X_L,X_E} \otimes \left(a\,|H\rangle_{XX_E} + e^{\delta(t)} b\,|V\rangle_{XX_E}\right)&\\
+ \frac{1}{2}|\phi^-\rangle_{X_L,X_E} \otimes \left(a\,|H\rangle_{XX_E} - e^{\delta(t)} b\,|V\rangle_{XX_E}\right)&\\
+ \frac{1}{2}|\psi^+\rangle_{X_L,X_E} \otimes \left(b\,|H\rangle_{XX_E} + e^{\delta(t)} a\,|V\rangle_{XX_E}\right)&\\
+ \frac{1}{2}|\psi^-\rangle_{X_L,X_E} \otimes \left(b\,|H\rangle_{XX_E} - e^{\delta(t)} a\,|V\rangle_{XX_E}\right)&
\end{aligned} \tag{8}$$

By performing a Bell state measurement on X_L and X_E (see previous section), the polarization of the output photon $|\psi\rangle_{XX_E}$ is defined instantaneously and can be related to the input polarization upon a unitary transformation

$$\begin{aligned} |\psi\rangle^{\phi^+}_{XX_E} &= \sigma_0(t)\,|\psi\rangle_{X_L} \\ |\psi\rangle^{\psi^+}_{XX_E} &= \sigma_x(t)\,|\psi\rangle_{X_L} \\ |\psi\rangle^{\psi^-}_{XX_E} &= \sigma_y(t)\,|\psi\rangle_{X_L} \\ |\psi\rangle^{\phi^-}_{XX_E} &= \sigma_z(t)\,|\psi\rangle_{X_L} \end{aligned} \tag{9}$$

where the time-dependent Pauli matrices describing the unitary transformation are

$$\sigma_0(t) = \begin{pmatrix} 1 & 0 \\ 0 & e^{\delta(t)} \end{pmatrix} \quad \sigma_x(t) = \begin{pmatrix} 0 & 1 \\ e^{\delta(t)} & 0 \end{pmatrix} \quad \sigma_y(t) = \begin{pmatrix} 0 & 1 \\ -e^{\delta(t)} & 0 \end{pmatrix} \quad \sigma_z(t) = \begin{pmatrix} 1 & 0 \\ 0 & -e^{\delta(t)} \end{pmatrix} \tag{10}$$

The complete density matrix of the output photon then reads

$$\rho_{XX_E} = \int_{t_{min}}^{t_{max}} P(t) \sum_B p_{|B\rangle}\, |\psi\rangle^B_{XX_E} \langle\psi|^B_{XX_E} (t) dt \tag{11}$$

where $P(t) = \frac{1}{T_{1_X}} e^{\frac{-t}{T_{1_X}}}$ is the probability distribution function over time and it is related to the temporal emission of an exciton with lifetime T_{1_X}. In the presence of a FSS, this term is important to evaluate the time-averaged fidelity to the expected entangled state (see Equation (6)), i.e., in the presence of a FSS, photons which come shortly after the excitation have a larger fidelity to the expected Bell state. Thereby, the temporal integration boundaries in Equation (11) provide a direct way to see how the density matrix evolves over time in the presence of a FSS. It is also important to note that in our comparison with the experiment we set the integration boundaries from 0 to ∞. This implies that we do not temporally post-select the emitted photons and, as a consequence, we do not resolve the time evolution of the density matrix but we simply see how the presence of a FSS affects the overall teleportation fidelity. In Equation (11), $p_{|B\rangle}$ is the probability to recognize a particular Bell state and it depends on the

quantum interference visibility V_{HOM}. In fact, while we arrange our set-up to detect $|\psi^-\rangle$ in our Bell state measurement, the non-perfect visibilities of two-photon interference implies that we may observe coincidences also from the other Bell state. In order to take into account this deviation from the ideal scenario, we assume that the probability to detect a $|\psi^-\rangle$ is depending linearly on the V_{HOM} as follows

$$p_{|\psi^-\rangle} = \frac{1}{4} + \frac{3}{4}V_{HOM} \tag{12}$$

On the other hand, this also means that as the visibility decreases, we must expect an increase in the probability of false detection originating from another (undesired) Bell state

$$p_{|\psi^+\rangle} = p_{|\phi^+\rangle} = p_{|\phi^-\rangle} = \frac{1}{4} - \frac{1}{4}V_{HOM} \tag{13}$$

The good agreement between theory and experiment confirms a posteriori that this linear approximation is indeed adequate. As Equation (11) allows us to calculate the density matrix of the output photon as a function of the relevant QD parameters (V_{HOM} and FSS) we can now calculate the teleportation fidelity as

$$F_T^{|\psi^-\rangle} = Tr[\rho_{XX_E}\sigma_y \, |\psi\rangle_{X_L} \, \langle\psi|_{X_L} \, \sigma_y^\dagger] \tag{14}$$

The average fidelity between a pure state and a mixed state is thereby understood when examining the system in the basis of the input state $|\psi\rangle_{X_L}$. It can be then easily seen that the fidelity will be equal to the matrix element $\rho_{X_L,X_L} := \langle\psi'_{X_L}|\rho_{XX_E}|\psi'_{X_L}\rangle$, where ψ'_{X_L} is the spin-flipped wavefunction of the input state. For more details we refer the interested reader to the reference given in the main text.

Density matrix reconstruction

We orient ourselves, without loss of generality, on the measurement of the density matrix for a diagonal input state, where we define the photon counts of our third-order correlation measure-

ments as

$$
\begin{aligned}
n_0 &= \frac{1}{2}(\langle H|\rho_{XX_E}|H\rangle + \langle V|\rho_{XX_E}|V\rangle) \\
n_x &= \langle L|\rho_{XX_E}|L\rangle \\
n_y &= \langle V|\rho_{XX_E}|V\rangle \\
n_z &= \langle D|\rho_{XX_E}|D\rangle
\end{aligned}
\tag{15}
$$

Here, the n_0 correlation counts serve as the normalization and are equivalent to the side peak correlation events individually evaluated for every single measurement, as explained in the main text. If we (at least) measure the third-order correlation for three orthogonal XX_E output states at the fixed diagonal input state, we can derive the corresponding Stokes vectors

$$
\begin{aligned}
S_0 &= \langle D|\rho_{XX_E}|D\rangle + \langle A|\rho_{XX_E}|A\rangle \\
S_x &= \langle D|\rho_{XX_E}|A\rangle + \langle A|\rho_{XX_E}|D\rangle \\
S_y &= \langle D|\rho_{XX_E}|A\rangle - \langle A|\rho_{XX_E}|D\rangle \\
S_z &= \langle D|\rho_{XX_E}|D\rangle - \langle A|\rho_{XX_E}|A\rangle
\end{aligned}
\tag{16}
$$

It is well known that these vectors allow to construct the polarization matrix via the Pauli spin matrices σ_i (see reference given in the main text)

$$
\rho_{XX_E}^{DA} = \frac{1}{2}\sum_{i=0}^{3}\frac{S_i}{S_0}\sigma_i \tag{17}
$$

These measurements adapted to the other input states allow us to reconstruct all the presented density matrices.

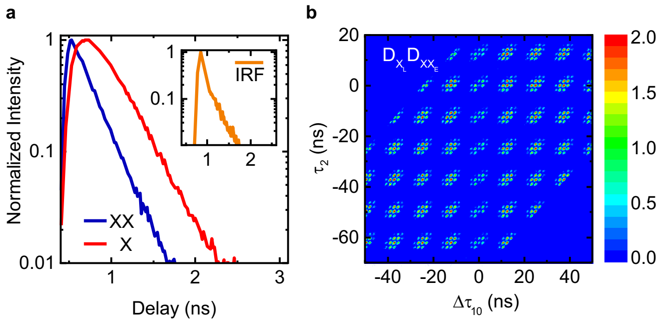

Fig. S1. Lifetime and full third-order correlation. (a) Measured lifetime of an arbitrary QD measured for both X and XX, respectively (Inset: Recorded instrument response function (IRF) used for the deconvolution of the experimental data). (b) Example of a normalized third-order teleportation correlation represented on large time scales for an arbitrary QD measured in the diagonal polarization base.

Table S1. Lifetimes of measured QDs. The resulting lifetimes of the X and XX transition for all studied QDs fitted by an exponential decay after deconvolution with the instruments response function.

QD [#]	XX [ps]	X [ps]
1	146(9)	268(16)
2	142(8)	259(10)
3	136(7)	255(8)
4	131(6)	271(15)
5	145(6)	273(10)

Table S2. Compiled experimental and theoretical results of all QDs under investigation. The measured two-photon interference visibility V_{HOM}, fine structure splitting FSS and corresponding entanglement fidelity f_E are given for each of the QDs. Together with the priorly shown lifetimes, the theoretical model is used to obtain the expected average teleportation fidelity f_T^{theory} and is compared to the experimental one $f_T^{exp.}$.

QD [#]	V_{HOM}[%]	FSS [μeV]	f_E [%]	$f_T^{exp.}$ [%]	f_T^{theory} [%]
1	65(2)	1.15(0.16)	93(0.3)	74.5(1.6)	74.0
2	62(3)	1.24(0.12)	91(0.3)	71.6(0.8)	72.5
3	57(4)	1.76(0.20)	86(0.3)	67.6(0.7)	68.3
4	57(4)	2.52(0.30)	82(0.3)	63.8(0.8)	64.6
5	58(4)	2.98(0.14)	78(0.3)	61.3(1.3)	63.2

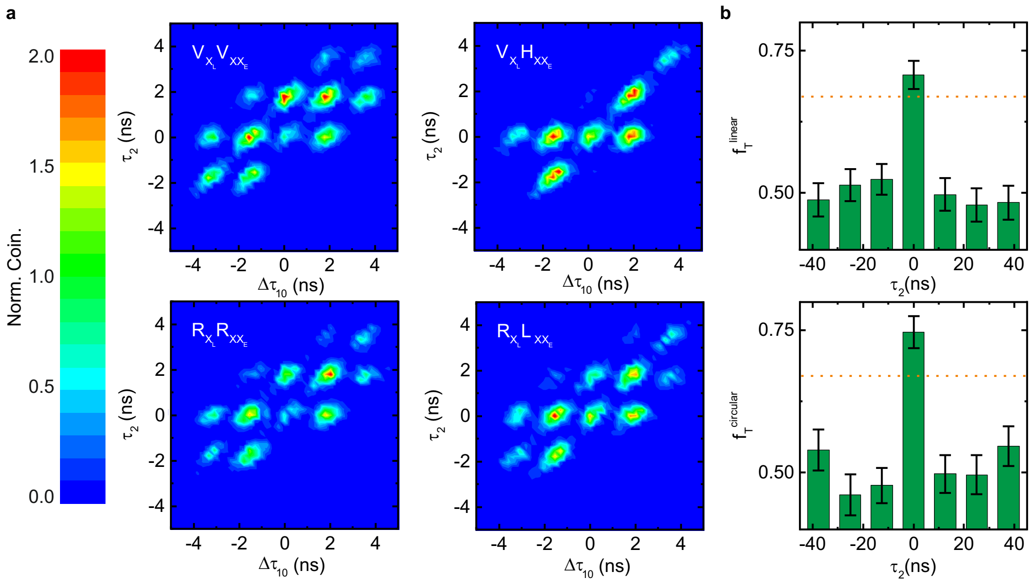

Fig. S2. Teleportation measurements on QD1. (a) Normalized third-order correlation of a V-polarized (top) as well as R-polarized (bottom) X input state for co-polarized (left) and cross-polarized (right) detection of XX photons. (b) Corresponding calculated values of the teleportation fidelity as a function of the systems duty cycle. We observe a fidelity of 71(3)% and 75(3)% for the linear and circular input state, respectively. The classical limit of teleportation is highlighted as a dashed-orange line.

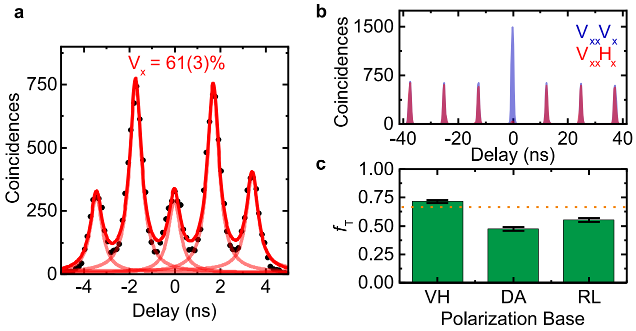

Fig. S3. Classical teleporter. (a) The two-photon interference measured in co-polarized configuration of an arbitrary chosen QD exhibiting a large FSS of 7.05(0.09) μeV. The depicted HOM visibility is the result from a fit with 5 Lorentzian peaks (bold red). (b) Cross-correlation of the non-entangled (time average) QD photons in the linear base. A correlation as high as 0.950(0.005) is observed after aligning the QD reference frame to the experimentally defined polarization frame with the aid of variable retarders. (c) The measured teleportation fidelity (identical experimental conditions as the investigated, entangled QDs) for differently polarized input states. Due to the intrinsic, classical correlation of the emitted QD photons a fidelity value of 72(1)% for linearly polarized photons, clearly above the classical limit, is observable. The average fidelity of 58(1)%, however, demonstrates the necessity to prove non-classical correlations in at least two basis to indicate true quantum teleportation.

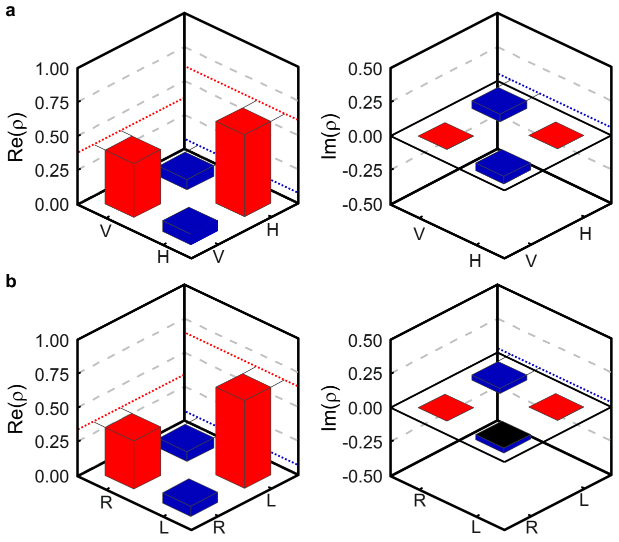

Fig. S4. Single-qubit density matrices for QD2. (a) Experimental real and imaginary part of the teleported single photon density matrix for a linear polarized and (b) circular polarized input state represented in the appropriate eigensystems, respectively. We extract $f_T^{linear} = 68.0(1.5)\%$ and $f_T^{circular} = 71.9(1.5)\%$.
